# The Effect of the Zonular Fiber Angle of Insertion on Accommodation

**DOI:** 10.3390/vision8030045

**Published:** 2024-07-23

**Authors:** Liying Feng, Barbara Pierscionek, Henk Weeber, Carmen Canovas Vidal, Jos J. Rozema

**Affiliations:** 1 Johnson & Johnson Surgical Vision, 9728 NX Groningen, The Netherlands; hweeber@its.jnj.com (H.W.); ccanovas@its.jnj.com (C.C.V.); 2 Visual Optics Lab Antwerp (VOLANTIS), Faculty of Medicine and Health Sciences, University of Antwerp, 2000 Antwerp, Belgium; 3 Faculty of Health Medicine and Social Care, Medical Technology Research Centre, Anglia Ruskin University, Chelmsford CM1 1SQ, UK; barbara.pierscionek@aru.ac.uk; 4 Department of Ophthalmology, Antwerp University Hospital, 2650 Edegem, Belgium

**Keywords:** zonular fibers, zonular angles, crystalline lens accommodation, finite element modeling

## Abstract

**Purpose:** With age, there is an anterior shift of the ciliary body in the eye, which alters the angle of zonular insertion in older eyes compared with younger eyes. This study aims to simulate lens accommodation with different zonular angles to consider the influence of zonular position on lens accommodative capacity. **Methods:** Models were constructed based on lenses aged 11, 29, and 45 years using a 2D axisymmetric structure that included a capsule, cortex, nucleus, and zonular fibers. The different zonular fibers were simulated by changing the position of the point where the zonular fibers connect to the ciliary body. The effect of the different zonular fiber insertion angles on the model shape and optical power was analyzed. **Results:** The models show that smaller angles made by zonular fibers to the surface of the lens lead to larger optical power changes with simulated stretching. When the models were stretched, and when varying the zonule angles, the optical power of the 11-, 29-, and 45-year-old models changed up to 0.17 D, 0.24 D, and 0.30 D, respectively. The effect of zonular angles on the anterior radius of curvature of the anterior surface varied by 0.29 mm, 0.23 mm, and 0.25 mm for the 11-, 29-, and 45-year-old models, respectively. **Conclusions:** Larger zonular fiber insertion angles cause smaller deformation and less accommodative change, while parallel zonules induce the largest change in lens shape.

## 1. Introduction

Accommodation is the ability to adjust the focus of the eye to obtain clear vision at different distances. The process involves the shape change of the lens. Multiple factors can affect the eye accommodative capacity. The lens becomes less flexible and loses its ability to change its shape with age; this is known as presbyopia [1] and is considered to be ubiquitous. The lens provides 30% of the refractive power of the eye for distance vision [2,3,4], and the contribution it adds for near vision alters in part because of the lenticular growth mode. The lens grows throughout life because of the continuous addition of new fiber cells that differentiate from the epithelial cells adjacent to the anterior capsule and increase the axial thickness of the lens at a rate ranging from 0.019 to 0.031 mm per year of life [5,6,7,8]. This growth leads to an increase in equatorial diameter with age [6]. In young eyes, the cortex has been found to have a higher value of Young’s modulus compared to the nucleus, and with age, the value of Young’s modulus in the nucleus apparently increases, surpassing the cortex in Young’s modulus [9,10,11,12]. However, care needs to be taken when using values of Young’s modulus as this can alter depending on the method of measurement used [12].

Furthermore, the ciliary body ring, as a whole, moves forward with age while significantly decreasing its diameter [13,14], suggesting that the zonular fibers gradually lose the ability to function. According to Farnsworth and Shyne [14], the distance between the zonular insertion point and the equator of the crystalline lens increases by 0.95 mm between the ages of 17 and 90 years [14]. Consequently, the forward movement of the ciliary body will alter the angles by which the zonular fibers are inserted into the lens.

Many studies have investigated the geometry changes of the crystalline lens during accommodation using Anterior Segment Optical Coherence Tomography (AS-OCT) and Ultrasound Biomicroscopy (UBM) [15,16] to obtain estimates for lens thickness, diameter, and curvature. Ex vivo analyses of extracted lenses in laboratory settings provide additional biomechanical data, allowing computational modeling through a finite element analysis (FEA). These measures, along with clinical studies assessing visual acuity and refractive error changes, collectively contribute to a better understanding of the mechanisms underlying accommodation [11].

Finite element modeling is especially suited to analyze the efficiency of crystalline lens accommodation as it allows for varying the shape and suspension of the crystalline lens. A number of studies applied finite element analysis on crystalline lens accommodation [17,18,19,20,21,22,23,24,25,26,27,28,29]. In the previous studies, the geometry was derived from measurements of the lens [2,13,14,30,31,32,33]. Burd et al. [23] applied shape parameters and mechanical properties from various studies [12,13,14,30,34,35], and the simulation methods of eye accommodation have been widely used in different studies of finite element modeling of the lens [21,24,27,28]. Burd’s work confirmed that the 45-year-old crystalline lens accommodated less efficiently than the 11- and 29-year-old lenses [23]. Wang et al. also simulated the crystalline lens at different ages and included several combinations of insertion angles for the zonular fibers [29,36], reporting that the lens had larger accommodative amplitude when the angles that the zonule makes with the lens surface were smaller. This suggests that zonular anatomical features and the material properties of the crystalline lens both play important role in the age-related decrease in accommodative amplitude, with geometry having the largest influence. The importance of the smaller angles of zonular insertion remains unclear. Theoretically, these angles could affect the efficiency by which the zonular fibers are able to transfer the stretching force to the lens, which would affect accommodation. This study presents analyses of 88 models with a wide range of zonular insertion angles to assess how these angles alter accommodative amplitude. Furthermore, to compare the sensitivity of accommodation to the zonular angles at different ages, three lens models were constructed using the same zonular angles, but with age-adjusted shape and material parameters.

## 2. Methods

To simulate accommodation with different zonular orientations, 2D axisymmetric models of crystalline lenses of three ages (11, 29, and 45 years old) were built in COMSOL Multiphysics (Version 6.0). The models included a capsule, cortex, nucleus, and zonular fibers based on the anatomic structure.

### 2.1. Geometry of Crystalline Lens and Zonular Fibers

**Figure 1:**
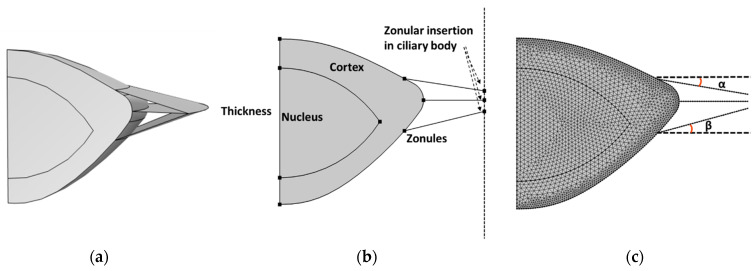
A 2D model of a 45-year-old lens to simulate accommodation with zonules modeled as the annular membrane (**a**,**b**) and the corresponding mesh (**c**). The model includes a cortex, nucleus, and capsule (a thin membrane covering the cortex). The zonules were inserted into the capsule and fixed to points on a vertical axis to represent the function of the ciliary body; the zonular insertion angles (α and β) were varied by moving the zonular insertion position on the ciliary body.

The dimensions of the crystalline lens were derived from various sources, including the fully accommodated lenses of 11-, 29-, and 45-year-old eyes as reported by Burd et al. [23], based on data from other studies [12,13,30,34]. The capsule, which provides structural support to the lens and maintains its shape, was modeled as a membrane element. The zonular fibers are delicate, thread-like structures composed of fibrillin and other proteins that are organized in three groups [14]: anterior, equatorial, and posterior zonules. The zonules were modeled as annular membranes which do not include the bending stiffness (shown in Figure 1a) surrounding the lens that extended to an outer diameter of 6.636 mm, 6.474 mm, and 6.330 mm, representing the diameter of the ciliary body in the accommodated state for 11-, 29-, and 45-year-old lenses, respectively. For simplicity, the function of the ciliary body was represented by three points in a fixed plane connected to the zonular fibers (Figure 1b), and for all zonules, the distance between the symmetry axis and the anchor point on the ciliary body was the same. The ciliary body motion was simulated by a linear displacement on the ciliary body side of the zonules [37]. The geometry was discretized as edge elements and free triangular elements. As shown in Figure 1c, 2150 and 1268 free triangular elements were used to create the cortex and nucleus, respectively, and 282 edge elements were used for each of the capsule and zonules. A mesh refinement study was conducted by discretizing the cortex and nucleus with 3418, 6961, and 11,776 elements. The results of the model thickness and anterior and posterior curvature did not change (as shown in an example in Table S1). Hence, the mesh shown in Figure 1c with 3418 elements was obtained.

### 2.2. Material Properties

The lenticular structure of the lens can be modeled with either a uniform or a radial distribution of the Young modulus in the cortex, leading to significant differences in stress distributions [29]. Pierscionek et al. measured the refractive index of human in vitro lenses over a wide age range by using X-ray Talbot interferometry [16], and this can been used as a basis for determining the distribution of Young’s modulus. To simplify the study, the cortex and nucleus were considered with uniform Young’s moduli as measured by Fisher [12], both with Poisson ratios of 0.49, and both were considered incompressible. Young’s modulus of the capsule was 0.35 MPa, and its Poisson ratio was 0.47 [35], as shown in Table 1.

### 2.3. Mechanical Modeling of Crystalline Lens Accommodation with Different Zonular Angles

For the 29-year-old lens model, the orientation of the anterior and posterior zonular fibers was varied in 2° increments between 0 and 10° and between 0 and 14°, respectively. The zonular angles were altered by shifting the insertion point on the ciliary body (Figure 1). A total of 48 combinations of zonular insertion angles were simulated in this study. In each case, the outer ends of the zonular fibers were stretched by 0.1 mm, 0.2 mm, 0.3 mm, and 0.36 mm to model decreases in accommodation. The stretch of 0.36 mm is the maximum amplitude of ciliary body movement given by Strenk et al. [13]. This power was calculated through the thick lens equation using radii of curvature estimated from the best fit circle applied to the coordinates of the lens surfaces over a central circular area with a diameter of 1.6 mm.

Similar models were built for 11- and 45-year-old lenses. Here, the anterior radius and posterior radius were limited to a range from 0° to 8° rather than 0° to 14°, to avoid the cross of anterior and posterior zonules. The 11-, 29-, and 45-year-old models were stretched for 0.46 mm, 0.36 mm, and 0.28 mm as the maximum amplitude of radial movement of the ciliary body reported by Strenk et al [13]. The surface power changes of the anterior and posterior capsule curvature caused by the stretch of the lens were analyzed, and the same was carried out for the changes in lens thickness and the total optical power. 

## 3. Results

### 3.1. Modelling of 29-Year-Old Lens Accommodation with 48 Zonular Angle Combinations

**Figure 2:**
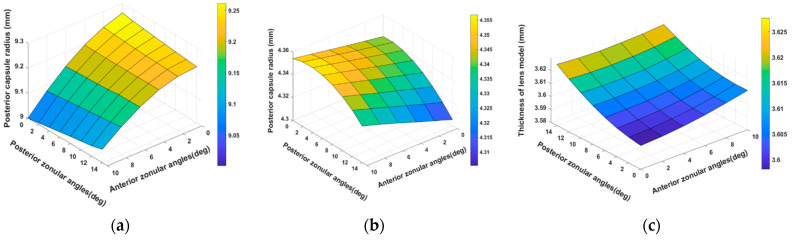
The difference in anterior radius of curvature (**a**) and posterior radius of curvature (**b**), and lens thickness (**c**) at the fully unaccommodated state of the 29-year-old model. Anterior zonular fibers’ angles varied between 0 and 14° and posterior zonular fibers’ angles varied between 0 and 14° in 2° steps.

Figure 2 shows the influence of zonular fiber orientations on simulated accommodation in the 29-year-old lens model, including the thickness change of the model and the radius of the fit to the anterior and posterior curvature for all the zonular angle combinations. Among all the zonular angle orientations, the largest value of the anterior radius of curvature was 9.26 mm. Similarly, the largest posterior radius of curvature in the unaccommodated models appeared when the anterior and posterior zonular angles were 0°, with a range between 4.33 mm and 4.35 mm over all the angles considered. Figure 2c shows the central lens thickness for the unaccommodated state. The model had the lowest sagittal thickness for near-parallel zonules, with a value of 3.60 mm, and the highest sagittal thickness, at 3.63 mm, for the largest zonular insertion angles considered. The model thickness and the change in anterior and posterior radii of curvature both contribute to the decrease in accommodative amplitude when the zonular fiber angles increase.

**Figure 3:**
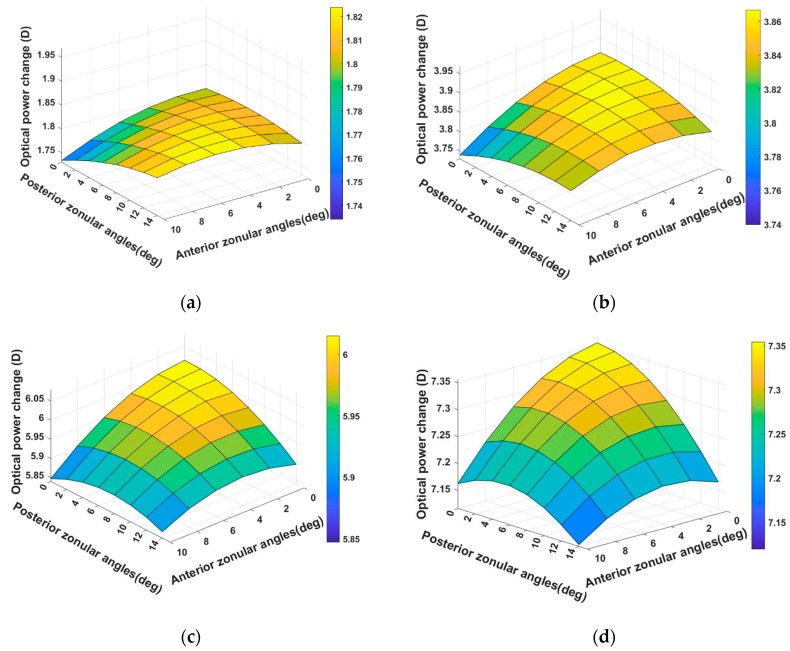
The difference in the optical power change of the four steps of the accommodation for the 29-year-old model. (**a**–**d**) The results when the ciliary body moved 0.1 mm, 0.2 mm, 0.3 mm, and 0.36 mm, respectively. Note that the figures have the same range on the vertical axis, but scales on the color bars.

The optical power change for the 29-year-old model reached its highest value when the zonules had the smallest angle (Figure 3). The largest and smallest optical power change for the fully unaccommodated model were 7.35 D and 5.12 D, respectively, indicating the scale of the influence of the insertion angle. When the model was stretched 0.1 mm, the optical power changed the most with the small anterior zonular angle and large posterior angle (Figure 3a). When the model was stretched 0.36 mm, the optical power changed the most with the small anterior and posterior zonular angles.

### 3.2. Comparison of Zonular Angles Effect on 11-, 29-, and 45-Year-Old Models

**Figure 4:**
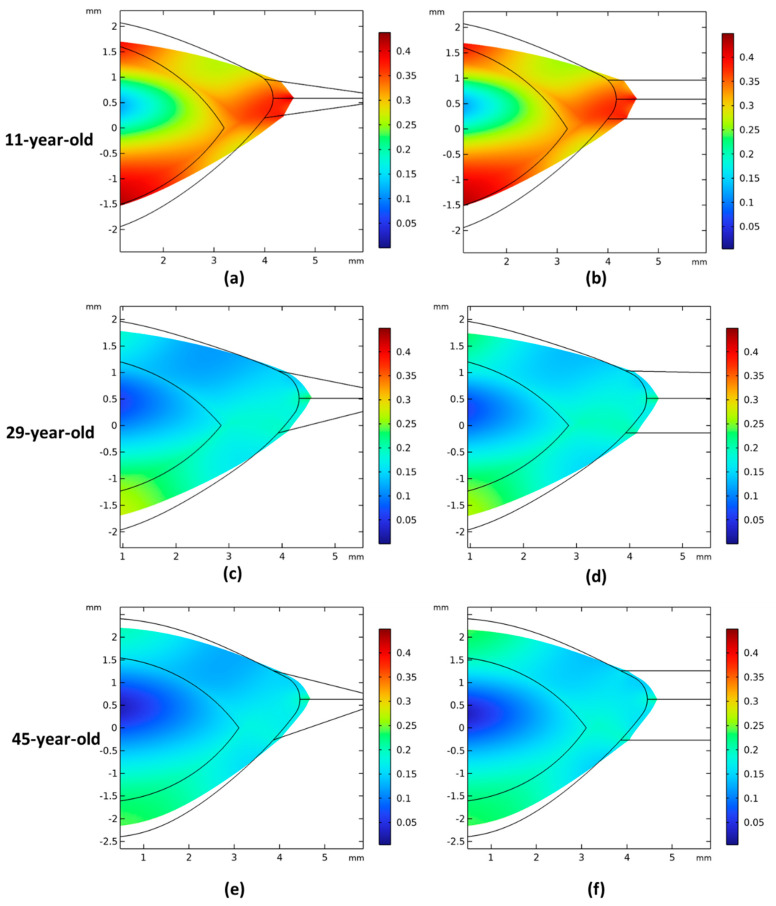
The displacement magnitude (mm) of 11-, 29-, and 45-year-old models during the (dis)accommodation; the zonules were stretched 0.46 mm, 0.36 mm, and 0.28 mm for 11-, 29-, and 45-year-old models. The left columns are the models with largest zonular angles; the right columns are the models with parallel zonules. (**a**) When zonular insertion angles have the largest value for 11-year-old models; (**b**) 11-year-old model with 0-degree zonular angles; (**c**) 29-year-old model with largest zonular angles; (**d**) 29-year-old model with 0-degree zonular angles; (**e**) 45-year-old model with largest zonular angles; (**f**) 45-year-old model with 0-degree zonular angles.

Figure 4 shows the displacement magnitude of the stretched models across three different ages, considering both the largest and smallest zonular angles. With increasing age, the models displaced less during accommodation. Both the anterior and posterior surfaces flattened significantly when zonular fibers stretched the lens, and the posterior surface displaced more than the anterior surface. Additionally, the cortex deformed more than the nucleus, with a distinct boundary between them. The lenticular deformation was larger when the stretching occurred through parallel zonula (Figure 4b,d,f).

**Figure 5:**
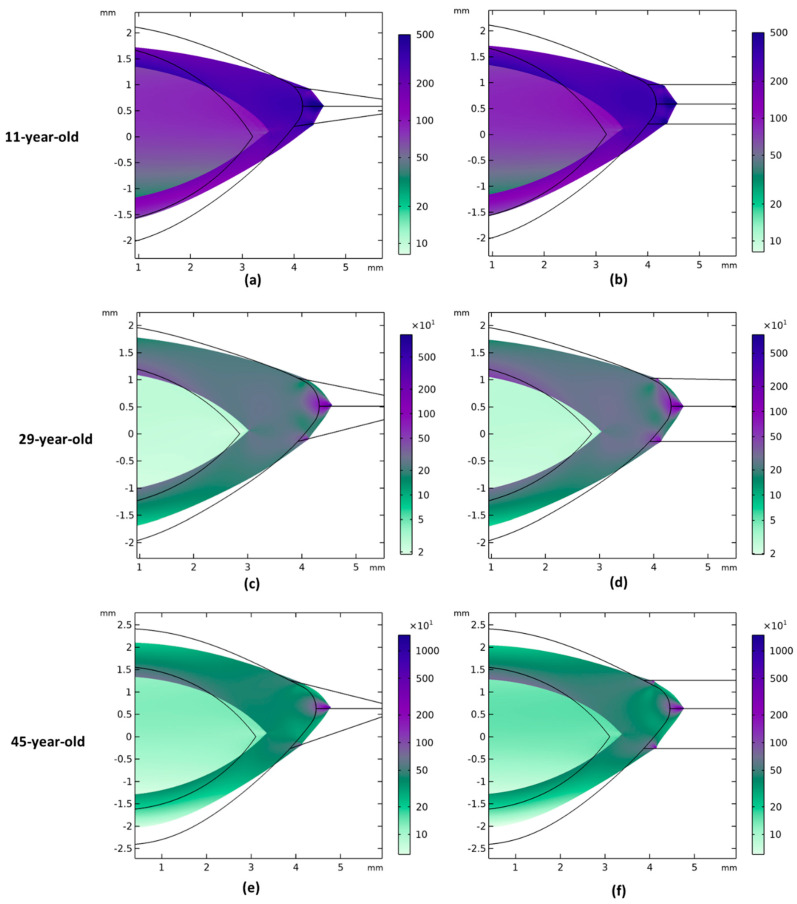
Stress distributions (von Mises stress in Pascal) for 11-, 29-, and 45-year-old lens models with large zonular angles and 0-degree zonular angles: (**a**) 11-year-old lens model with largest zonular angles; (**b**) 11-year-old lens model with 0-degree zonular angles; (**c**) 29-year-old lens model with largest zonular angles; (**d**) 29-year-old lens model with 0-degree zonular angles; (**e**) 45-year-old lens model with largest zonular angles; (**f**) 45-year-old lens model with 0-degree zonular angles. The range of color bar scale was based on the largest and smallest value of von Mises stress. The black frame is the lens model geometry before the lens was stretched by zonular fibers.

The von Mises stress distributions of the models are shown in Figure 5. Models with different zonular angles had similar stress distributions, and the posterior part of the lens model experienced less stress than the anterior part. There is an obvious difference in stress distribution at the border between the cortex and the nucleus. The highest stresses can be found where the zonules are attached to the model, while the lowest stresses are at the posterior pole of the lens. Younger lens models experienced smaller stresses than the older lens models.

**Figure 6:**
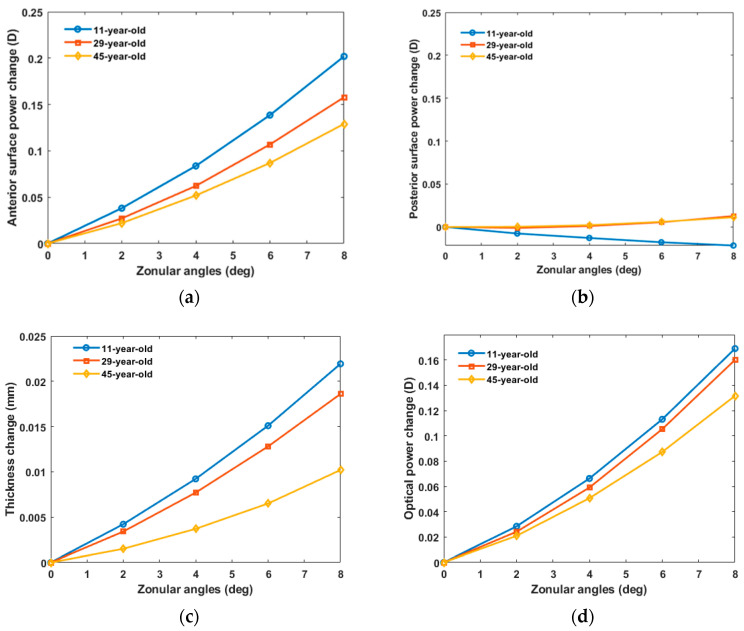
The effect of changes in zonular angles on crystalline lens model surface power, lens thickness, and optical power for lenses of three ages (11, 29, 45 years). Values are presented as changes relative to the parallel zonular fibers (angles of 0°). Anterior and posterior zonular angles are the same. (**a**) Change in anterior surface power; (**b**) change in posterior surface power; (**c**) change in crystalline lens model thickness; (**d**) optical power change.

The effect of the zonular angles on the anterior and posterior radii of curvature at different ages is shown in Figure 6. For each age, the zonular angles were identical for anterior and posterior zonules. The values shown in the figure are the surface power, thickness, and optical power values minus the 0° zonular angle. The anterior surface power, lens thickness change, and optical power increased when larger zonular angles increased. However, the posterior surface power showed only very minor changes by comparison. The lenses became less sensitive to the zonular angle changes with age. Compared with posterior surface power, the anterior surface power was more influenced by zonular angles.

## 4. Discussion

This study developed a series of models based on the geometry of a study of finite element simulation of the accommodation [23] of 11-, 29-, and 45-year-old lens models, including the capsule, cortex, and nucleus. Lens thickness at rest was found to be lower for parallel zonula and higher for larger angles. The accommodative range also decreased for larger zonular angles.

For the 29-year-old model, when the anterior and posterior zonule angles were parallel (0°), the lens thickness decreased by 0.53 mm after the lens model was stretched from a fully accommodated state. When the zonular fibers formed an angle of 10° and 14° for the anterior and posterior zonules, respectively, the thickness change of the lens model after the (dis)accommodation was 0.50 mm. Some studies determined the lens geometry for different levels of accommodation [6,38,39]. Dubbelman and Martinez-Enriquez’s reported a thickness change of +70 µm per diopter (µm/D) of accommodation in a 29-year-old lens. Other studies reported a thickness change of +36 µm/D to +44 µm/D [40,41]. These results are smaller than +120.8–124 µm/D in this study, which may be due to differences in the mechanical properties of the models.

When the lens was fully stretched, the results of the displacement magnitude in this study (Figure 4) showed that the posterior lens had larger displacement than the anterior lens; meanwhile, the posterior radius of curvature had less change than the anterior radius of curvature after the stretch of the lens. In the study of Dubbelman et al. [31], the movement of the anterior lens surface was, on average, five times greater than the posterior. The discrepancy was possibly because the model in this study did not consider the ligament of Wieger, which might dampen the posterior curvature change during accommodation [18].

The anterior and posterior radii of curvature of the fully accommodated lens considered in this study were 9.05 mm and 4.33 mm, respectively, based on in vivo measurements by Brown et al. [30]. When the lens was fully stretched, the anterior curvature radius was 9.00–9.26 mm depending on the zonular insertion angle, while the posterior curvature radius was 4.33–4.35 mm. Other studies reported different values, such as Fisher, who measured 24 lenses and reported that the mean anterior radius of curvature of a 43-year-old accommodated lens in vitro was 9.2 mm [42]. Dubbelman et al. [31] used Scheimpflug images to determine the lens shape changes in accommodating eyes at different ages in vivo. The anterior radius of curvature at maximum accommodation over a 3 mm zone was about 9 mm, while the posterior radius was 5.3 mm (data read from a graph). The possible reason for the difference between lens radii of the model and those in the literature may lie in difference in age and refractive error.

Wang et al. reported changes in the radius of curvature and central optical power caused by several different zonular insertion angles [29]. They used an anterior radius of curvature of the unaccommodated crystalline lens model of 10.6 mm for a 35-year-old eye, and a posterior radius of 6.7 mm, considerably flatter than the values of 9.05 mm and 4.33 mm used here, and worked at larger zonular angles. Compared with Wang’s study, our model had a smaller change in posterior radius of curvature during accommodation (Table 2).

Glasser et al. discussed the age-related changes in the optical properties of the human crystalline lens and their relationship with the development of presbyopia, based on ex vivo lenses in an 8-arm stretching device [43]. Their study found that the accommodative amplitude reduces from about 8D for a 29-year-old lens to about 2D for a 45-year-old lens, and that after the age of 58 years, the lens loses its ability to accommodate. Pierscionek investigated the in vitro alteration of human lens curvatures by radial stretching [44] and found that 27-year-old and 46-year-old eyes did not show large differences in the posterior radius of curvature change during stretching. In this study, the changes in the posterior radius of curvature change were all lower than the change in the anterior radius of curvature for the three ages (Figure 6a,b), which aligns with previous studies [31,44].

In the study of Wang et al., the anterior zonules varied from 10° to 26°, while the posterior zonules varied from 24° to 40°. The central optical power difference caused by the zonular insertion angles in their study was around 5 D, while in this study, the optical power affected by zonules was 0.24 D for the 29-year-old lens model. The reason for this difference may be the initial geometry of the crystalline lens models and the variation in the initial configuration of the anterior and posterior curvature, both of which can lead to different pressure distribution on the anterior and posterior areas of the lens. Furthermore, the elastic modulus of the present study (cortex 3.42 kPa and nucleus 0.55 kPa) is larger than that of Wang’s study (cortex 0.82 kPa and nucleus 0.04 kPa), which will reduce lens deformation and its sensitivity to the zonular fiber orientations.

Changes in zonular angles affect young lenses more than older lens (Figure 6), which can be attributed to the age-related increase in the Young’s modulus of the lens. For posterior surface power, the results effected by zonular angle alteration was very limited, and the power change is not clinically significant. Wang et al. [29] reported that when the anterior and posterior zonular angles increased to 8°, the anterior radius of curvature of a 16-year-old lens became steeper by 1.2 mm and by 1.9 mm for a 62-year-old lens. The posterior radius of curvature became steeper by 0.6–0.7 mm, respectively. Their study showed a larger age-related difference than this study, which may be due to differences in lens geometry, material properties, and the choice of zonular angles.

There was a clear boundary between the cortex and nucleus (Figure 5), which concurred with a previous study reporting an abrupt difference in the stiffness of the cortex and nucleus [45], but may not necessarily reflect the situation in the living lens. There were also high levels of stress seen at the zonule attachment points. Anatomically, the zonular fiber insertions may be spread over a wider area and depth than the modeled attachment to a single discrete point, which would spread out the stress over a wider area.

This study has some limitations, as the model simplified the zonular attachment between the ciliary body and crystalline lenses by distributing the zonular fibers over a narrow region of the capsule and modeling them as three annular sheets. Moreover, there have been few studies on zonular fiber insertion in vivo [14,46], making it difficult to validate the simulation results. In addition, there are differences in the assumed material properties of the lens between studies [30,42,47,48] that would also lead to variations in results. Modeling is a generic approach, and the accuracy of the results is highly dependent on the input parameters used in the models. These input parameters, in turn, are influenced by the specific methods and conditions under which they were measured. Consequently, variations in measurement techniques and experimental conditions can introduce uncertainties and limitations to the model’s predictive capabilities.

In summary, this modeling suggested a larger optical power change with a decrease in the zonular angle. The anterior surface curvature was more sensitive to the zonular fiber angle change than the posterior surface curvature. The angles of zonular fibers contribute to accommodation, and alterations in the orientation or alignment of zonular fibers may affect accommodative capacity.

## Figures and Tables

**Table 1:** The material properties of the capsule, cortex, and nucleus for three ages.

	Age	Young’s Modulus (pa)	Poisson’s Ratio
Capsule	11	0.73 × 10^6^	0.47
	29	1.27 × 10^6^	
	45	1.45 × 10^6^	
Cortex	11	1875	0.49
	29	3417	
	45	3980	
Nucleus	11	569.5	0.49
	29	547.4	
	45	996.6	

**Table 2:** Comparison between Wang et al. and present study. Table 1 comparison between Wang et al. and present study. Radius change is the difference between the radius of the crystalline lens models before and after accommodation (optical power change data of Wang’s study was read from figure).

Study	Angles [Anterior, Equatorial, Posterior]	Anterior Radius Change (mm)	Posterior Radius Change (mm)	Optical Power Change (D)
Wang et al. [29]	[10, 0, 24]	2.4	1.3	3
	[18, 0, 32]	0.7	0.6	–
	[26, 0, 40]	−0.7	0	–
This study	[10, 0, 14]	3.2	0.48	7.12
	[0, 0, 0]	3.4	0.49	7.35

## Data Availability

The datasets used and/or analyzed during the current study are available from the corresponding author on reasonable request.

## Supplementary Materials

The following supporting information can be downloaded at https://www.mdpi.com/article/10.3390/vision8030045/s1, Table S1. The lens thickness and optical power change results of the mesh refinement study.
