# Supplementary material for: The Effect of the Zonular Fiber Angle of Insertion on Accommodation
Source: Vision (Basel). 2024 Jul 23;8(3):45. doi: 10.3390/vision8030045 (PMC11348028; doi:10.3390/vision8030045)
Supplement: Supplementary file 1 [file vision-08-00045-s001.zip › vision-3058066-supplementary.pdf]

vision

Supplementary Material: The Effect of the Zonular Fiber Angle of Insertion on Accommodation

Liying Feng, Barbara Pierscionek, Henk Weeber, Carmen Canovas Vidal and Jos J. Rozema

Table S1. This table shows the results of 45-year-old lens when the ciliary body stretched the lens for 0.2767 mm. The mesh refinement study of the models. Three sizes of the element were applied.

Anterior zonular angle (°)	Posterior zonular angle (°)	Number of the elements	Thickness (mm)	Optical power change (D)
0	0	3418	4.3588	4.0408
0	0	6961	4.3588	4.0408
0	0	11776	4.3588	4.0408
2	2	3418	4.3603	4.0199
2	2	6961	4.3603	4.0199
2	2	11776	4.3603	4.0199
4	4	3418	4.3625	3.9901
4	4	6961	4.3625	3.9901
4	4	11776	4.3625	3.9901
6	6	3418	4.3653	3.9535
6	6	6961	4.3653	3.9535
6	6	11776	4.3653	3.9535
8	8	3418	4.3690	3.9091
8	8	6961	4.3690	3.9091
8	8	11776	4.3690	3.9091
